# Composite eosinophilic solid and cystic renal cell carcinoma and clear cell renal cell carcinoma: a rare case report and literature review

**DOI:** 10.1186/s12894-024-01542-4

**Published:** 2024-07-30

**Authors:** Xian Zhang, Lin Li, Lisha Wang, Mengxing Yu, Dongdong Zhang

**Affiliations:** 1 Department of Radiology, Xiangyang No. 1 People’s Hospital, Hubei University of Medicine, Xiangyang, 441000 China; 2 Department of Cardiology, Xiangyang No. 1 People’s Hospital, Hubei University of Medicine, Xiangyang, 441000 China; 3 Department of Pathology, Xiangyang No. 1 People’s Hospital, Hubei University of Medicine, Xiangyang, 441000 China; 4 Department of Oncology, Xiangyang No. 1 People’s Hospital, Hubei University of Medicine, Jiefang Road No.15, Xiangyang, 441000 Hubei China

**Keywords:** Eosinophilic solid and cystic renal cell carcinoma, Clear cell renal cell carcinoma, Renal composite tumors, Surgery

## Abstract

**Background:**

Eosinophilic solid and cystic renal cell carcinoma (ESC-RCC) is a novel subtype of renal cell carcinoma characterized by its relatively low incidence and indolent behavior. We report a rare case of ESC-RCC concurrent with clear cell renal cell carcinoma (ccRCC) in a single kidney.

**Case presentation:**

A 48-year-old male, was found to have a mixed echogenic mass in the left kidney during a physical examination. He has no history of hematuria and flank pain. An abdominal CT scan revealed a 3.0 * 1.9 * 2.5 cm^3^ mass with unclearly bordered at the lower pole of the left kidney. Abdominal MRI showed two nodules of different sizes in the left kidney, suggesting the possibility of a tumor. The patient underwent a subtotal nephrectomy, and the postoperative pathological results indicated ESC-RCC combined with ccRCC. The patient recovered well without tumor recurrence during the 12-month follow-up.

**Conclusion:**

We reported a case of renal composite tumors, comprising the rare ESC-RCC and the more common ccRCC. Imaging combined with postoperative pathological examination is crucial for the definitive diagnosis of these rare tumors.

## Introduction

Eosinophilic solid and cystic renal cell carcinoma (ESC-RCC) is a novel subtype of RCC characterized by solid and cystic structures and eosinophilic tumor cells. It was first reported in 2016 and was officially listed as a new subtype of renal cell carcinoma in the 2022 World Health Organization (WHO) classification of renal tumors. ESC-RCC accounts for approximately 0.2% of all renal tumors and only less 70 cases were reported worldwide [1].

Renal composite tumors are defined as the presence of two distinct pathological components originating from the kidney, with a significant confluent zone and separated by normal renal parenchyma [2]. Renal composite tumor is a rare clinical phenomenon, with most clinical reports being of composite clear cell renal cell carcinoma (ccRCC) and papillary RCC, the two most common types of kidney tumors [3]. Since ccRCC has been separately classified as a distinct subtype of RCC, the composite ESC-RCC and ccRCC has not been reported yet. This study first reports a rare case of ESC-RCC co-occuring with ccRCC.

## Case presentation

**Fig. 1:**
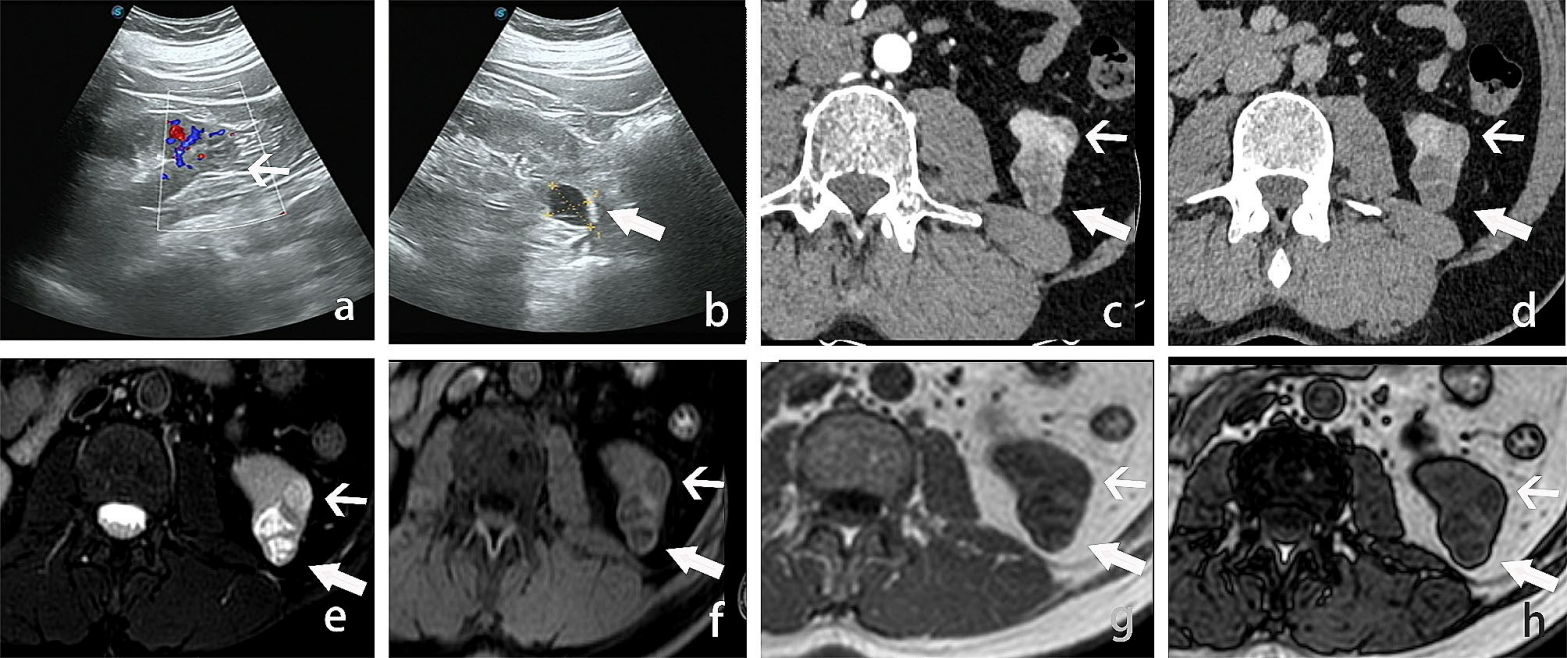
Imaging findings of composite renal tumor. **a-b**: Ultrasound images demonstrated a solid echoic nodule (lesion 1) at the lower pole of the left kidney (fine arrow), with CDFI indicating rich blood vessels. Another cystic echoic nodule (lesion 2) is also seen (coarse arrow). **c-d**: Enhancement CT showed that the anterior lesion (lesion 1, fine arrow) exhibited significant enhancement during the arterial phase and washout during the delayed phase. The posterior lesion (lesion 2, coarse arrow) exhibited mild and homogeneous enhancement in both the arterial and delayed phases. **e-h**: MRI images revealed that Lesion 1 (fine arrow) displayed slight hyperintensity on T2WI, while Lesion 2 (coarse arrow) showed heterogeneous hyperintensity. Both lesions appeared hypointense on T1WI and in/out phase

**Fig. 2:**
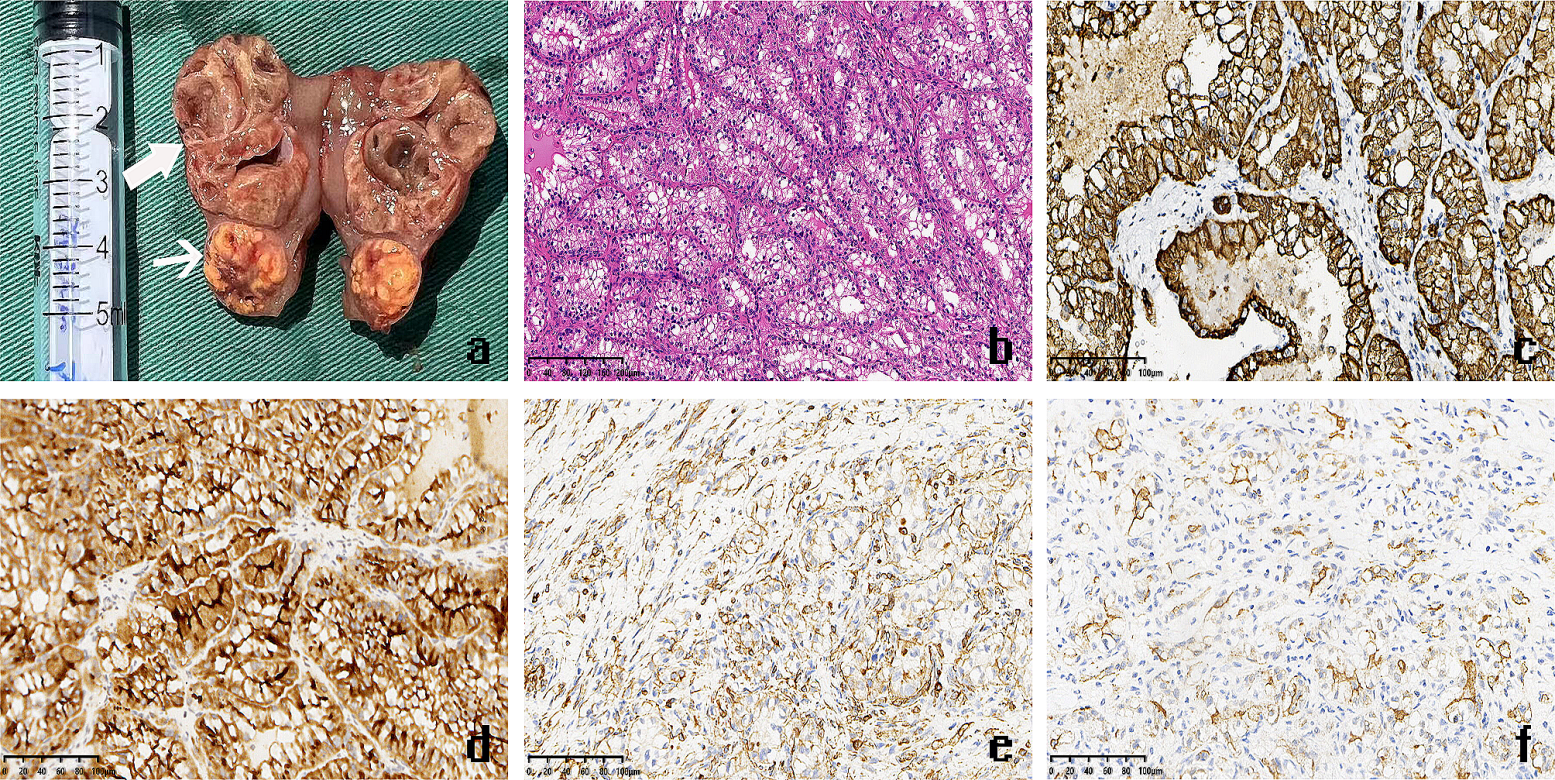
The gross specimen and the pathological findings of ccRCC. **a**: The surgical excision of the gross specimen reveals two masses of different colors and sizes. Lesion 1 appears solid (fine arrow), while Lesion 2 exhibits multiple cystic areas (coarse arrow). The boundary between the two masses is very clear. **b**: H&E staining analysis reveals optically clear cytoplasm, prominent cell membranes, and a vascular network of tumor cells at 100× magnification. **c-f**: IHC indicates the tumor cells were positive for CA9 (**c**), CD10 (**d**), vimentin (**e**), and EMA (**f**) at the magnification of ×200, suggesting the pathological diagnosis of ccRCC

**Fig. 3:**
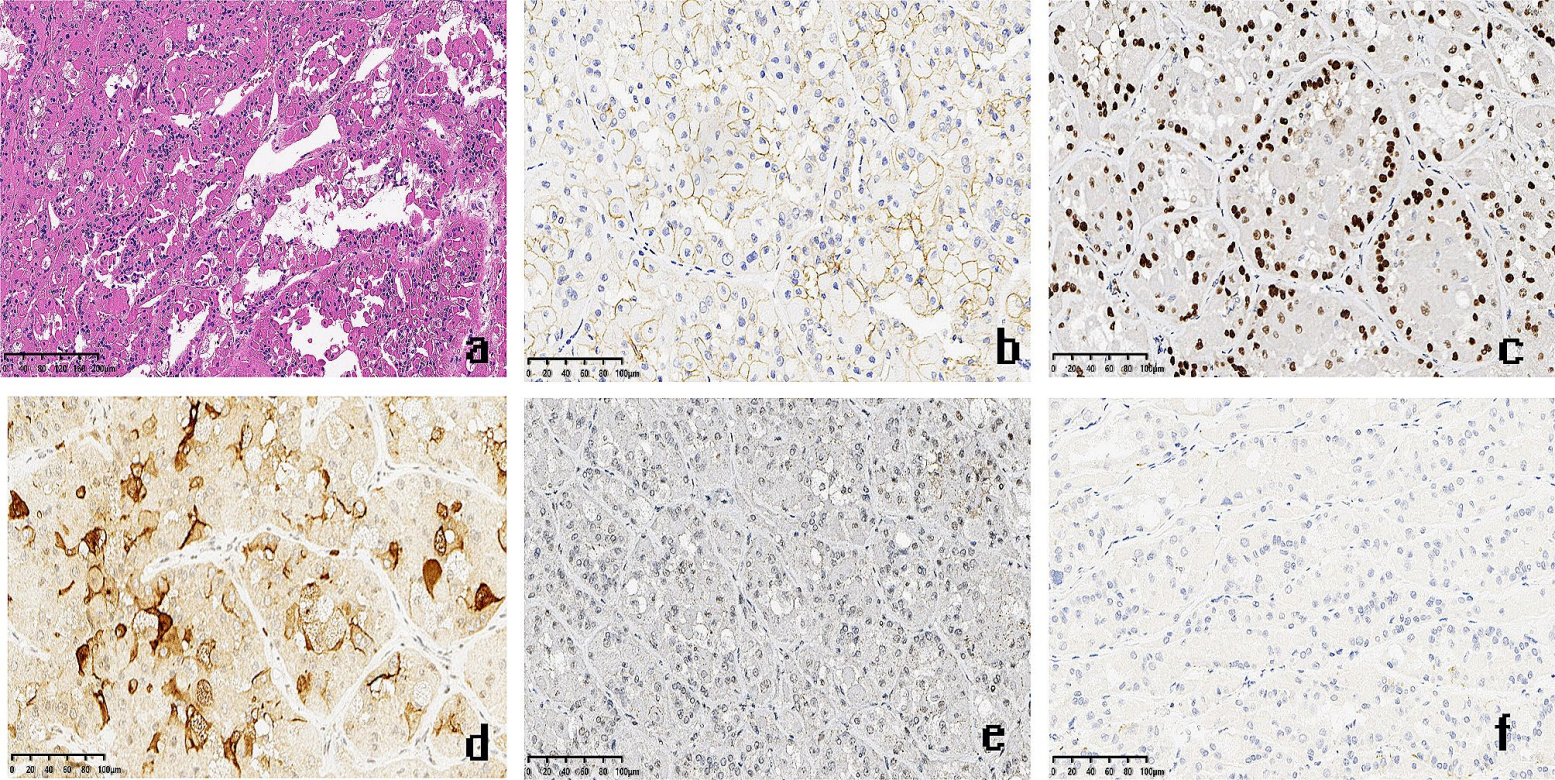
Pathological examination of ESC-RCC. **a**: H&E staining reveals that the tumor contains cystic areas, lined by eosinophilic cells with abundant cytoplasm. **b-f**: IHC indicates that the tumor cells were positive for CK20 (**b**), PAX8 (**c**) and CD10 (**d**), but negative for CA9 (**e**) and CK7 (**f**), suggesting the diagnosis of ESC-RCC

A 48-year-old male patient was found to have an abnormal mixed echo in the lower pole of the left kidney during a physical examination ultrasound (Fig. 1a-b). The patient exhibited no symptoms of hematuria, urinary frequency, or urgency, and no other relevant urinary symptoms were noted. He was generally healthy with no significant medical history and family history. The patient was admitted for further examination. The CT scan revealed two iso-dense nodules with irregular shapes and indistinct borders in the lower pole of the left kidney. Lesion 1, located in the anterior aspect of the left kidney, demonstrated significant enhancement in the arterial phase and washout in the delayed phase; Lesion 2, situated in the posterior aspect, exhibited mild and homogeneous enhancement in both the arterial and delayed phases (Fig. 1c-d). MRI imaging revealed two nodules in the lower pole of the left kidney, with inconsistent signal characteristics. Lesion 1, located anteriorly, displayed a slightly elevated T2 signal, while Lesion 2, situated posteriorly, showed hyperintensity. In-phase and Opposite-phase imaging indicated no evidence of fat components. Diffusion-weighted imaging revealed slightly high signal intensity in Lesion 1 and equal signal intensity in Lesion 2 (Fig. 1e-h). Based on the results of the imaging examination, a renal tumor is highly suspected. The patient then underwent a retroperitoneoscopic partial nephrectomy of the left kidney, the tumor and surrounding renal tissue were completely removed. The surgery removed a tumor with an overall volume of approximately 3.5 *3.0 *3.0 cm2, consisting of two masses of different colors and sizes, with indistinct boundaries between them at the connecting point (Fig. 2a). Morphological examination and immunohistochemical (IHC) analysis were conducted separately on the two lesions. Lesion 1, the size is approximately 1.2 * 0.5*0.2 cm^3^. H&E staining analysis reveals optically clear cytoplasm, prominent cell membranes, and a vascular network of tumor cells (Fig. 2b). IHC indicates the tumor cells were nuclear positive for CA9, CD10, vimentin, and EMA, which suggest the pathological diagnosis of ccRCC (Fig. 2c-f). Lesion 2 measures approximately 2.5*2.0 *1.3 cm^3^. H&E staining reveals that the tumor contains cystic areas, lined by eosinophilic cells with abundant cytoplasm (Fig. 3a). IHC indicated that the tumor cells were positive for CK20, CD10 and PAX8, but negative for CK7 and CA9, suggesting the diagnosis of ESC-RCC (Fig. 3b-f). Accordingly, the final diagnosis was renal composite ESC-RCC and ccRCC. The postoperative TNM staging of the patient was T1aN0M0, staging I, low risk (University of California, Los Angles Integrated Staging System). The patient recovered well after surgery, with no tumor recurrence or progression observed during the one-year follow-up.

## Discussion

ESC-RCC is an under-recognized, newly identified RCC subtype with unique clinical manifestations and molecular immune phenotypes. Before 2010, ESC-RCC was initially defined as RCC associated with tuberous sclerosis complex (TSC) because the majority of ESC-RCC cases had been identified in adult women with TSC [4]. However, since 2016, sporadic cases of ESC-RCC have been reported in patients without TSC [5]. Subsequent studies have shown that most cases of ESC-RCC do not occur concurrently with TSC; only approximately 10% of patients have both conditions simultaneously [6]. The tumor was previously classified as “unclassified renal tumors with oncocytic or eosinophilic granular cell morphology” or “unclassified renal cell carcinoma” until it was recognized as a rare novel type of RCC in 2022 [7, 8].

ESC-RCC tends to occur in middle-aged women, but it can also affect people of all ages and men. The male-to-female ratio of the tumor is 1:1.2–1.7, and the age range of onset is between 14 and 79 years old [9]. ESC progresses indolently, and many patients do not exhibit obvious clinical symptoms, often being discovered incidentally. Nearly 89% of patients receive a diagnosis in the early stages (stage T1) [5, 10]; however, scattered reports also exist of cases with distant metastases [7]. The incidence of ESC-RCC, renal collision, and composite tumors is relatively low. To the best of our knowledge, this is the first reported case of a composite ESC-RCC and ccRCC to date.

ESC-RCC is typically well-defined, unifocal and solitary tumor with small size. Grossly, the tumor appears yellow, gray, or brownish in color, is well delineated and encapsulated, with numerous cystic and solid areas internally [11]. Microscopically, the cystic wall is lined with hobnail-like tumor cell, while the solid areas are densely populated with tumor cells featuring eosinophilic cytoplasm; foam cells and lymphocytes are present in the stroma [12].

Imaging examinations, including MRI, hold diagnostic value for ESC-RCC. Yi et al. summarized the MRI characteristics and classified ESC-RCC into three types: (a) Type I shows a characteristic “lotus root-like” appearance; (b) Type II displays a “honeycomb-like” appearance with thick-walled cystic tumors and nodules on the walls; (c) Type III presents as a solid tumor without cystic, necrotic, or hemorrhagic areas [13]. Our case is consistent with the characteristics of type 2 ESC-RCC. Undoubtedly, pathology is the gold standard for diagnosing ESC-RCC. A typical immunophenotype for ESC-RCC is characterized by focal or diffuse CK20 positivity paired with CK7 negativity, distinguishing it from other eosinophilic tumors of the kidney [11]. Since mutations in *TSC*, including *TSC1* and *TSC2*, are present in 85% of ESC-RCC cases, these mutations can be important molecular characteristics for ESC-RCC [11]. It is important to emphasize that *TSC* mutations cannot be used as a diagnostic criterion for ESC-RCC. Some patients exhibit copy number gains or losses and loss of heterozygosity in other chromosomes, which are related to the regulation of mTOR signaling pathway [11]. Mutations in *TSC1* or *TSC2* genes result in a loss of function of the hamartin/tuberin complex, leading to the constitutive activation of mTORC1. This indicates that mTOR inhibitors may be used to treat TSC-related tumors by inhibiting mTORC1, especially for ESC-RCC with *TSC* mutation. The limitation of this study is that we did not perform genetic testing; therefore, we do not know whether the patient has any relevant genetic mutations. The appearance of renal composite tumors is atypical, and they often share common driver genetic mutations. Nearly 95% of ccRCCs are associated with *3p* deletions and somatic inactivating mutations of the von Hippel-Lindau gene [14]. However, solitary ESC-RCCs often exhibit *TSC* mutations. Our case may help promote in-depth research on the pathogenic genes and metastatic behavior of this type of malignancy.

The treatment principles for ESC-RCC refer to the standards for renal cancer treatment [15]. For localized kidney cancer, surgery is the mainstay treatment. Patients who cannot tolerate surgery may be closely observed or undergo ablation or stereotactic radiotherapy. For locally advanced kidney cancer, surgery or systemic drug therapy can be chosen based on the patient’s condition. For patients with newly diagnosed metastatic kidney cancer, systemic drug therapy, including targeted therapy and immunotherapy, can be chosen. According to the National Comprehensive Cancer Network Guidelines for Kidney Cancer, the preferred systemic treatment for non- ccRCC is tyrosine kinase inhibitors (TKIs) [16]. However, some studies indicated that for patients with distant metastasis, the efficacy of mTOR inhibitors is superior to that of TKI [17]. Other recommended regimens include TKI plus mTOR inhibitor, PD-1 inhibitors alone, or in combination with TKIs. However, there are few reports in the literature regarding its efficacy in ESC-RCC.

Due to the indolent biological characteristics of ESC-RCC, 90% of patients are diagnosed at either stage T1 or T2 [18]. These patients can be cured through partial nephrectomy. Approximately 5-10% of patients with ESC-RCC experience distant metastasis, commonly occurring in the lungs, liver, and bones [11, 13]. Existing literature reports that only one reported case of death among patients with distant metastasis [1, 13]. Therefore, the overall prognosis for ESC-RCC is generally favorable.

In conclusion, we reported a rare case of composite ESC-RCC and ccRCC who recovered well after partial nephrectomy. We detailed the imaging characteristics of this newly reported renal composite tumor and summarized the diagnosis and treatment of ESC-RCC. Our case not only enriches the data bank of renal collision composite tumors, but also deepens our understanding of this emerging type of RCC.

## Data Availability

The clinical data supporting the conclusions of this manuscript will be made available by the authors.
